# Fezakinumab Alleviates Cigarette Smoke–Induced COPD by Suppressing the JAK1/STAT3 Pathway in Mice

**DOI:** 10.1155/carj/3204642

**Published:** 2025-11-29

**Authors:** Yuwei Wang, Li Jin, Erche Yang, Xiaoqun Niu, Jianchuan Mao, Chaoqun Yuan, Yong Wang, Bo You, Lan Liu, Yanling Chai

**Affiliations:** ^1^Department of Respiratory and Critical Care Medicine, People's Hospital of Yuechi County, Yuechi, Sichuan 638300, China; ^2^Department of Anesthesiology, People's Hospital of Yuechi County, Yuechi, Sichuan 638300, China; ^3^Department of Respiratory and Critical Care Medicine, Dali Bai Autonomous Prefecture People's Hospital, Dali, Yunnan 671000, China; ^4^Department of Respiratory and Critical Care Medicine, The Second Affiliated Hospital of Kunming Medical University, Kunming, Yunnan 650101, China

**Keywords:** AG490, apoptosis, fezakinumab, IL-22,COPD, JAK/STAT pathway

## Abstract

**Background:**

Elevated Th-22 cells and IL-22 are linked to chronic obstructive pulmonary disease (COPD); however, their mechanisms are not fully elucidated. This study aimed to evaluate the therapeutic effects of AG490 and fezakinumab in a cigarette smoke–induced COPD mouse model.

**Methods:**

Cigarette smoke–induced COPD mice were divided into Air, CS, AG490, and Fezakinumab groups. We assessed BALF TH-22 cell levels, IL-22 levels and lung tissue (lung tissue were taken from the same anatomical site of the left lower lobe) histopathological changes, IL-22R1 expression, and protein expression of Jak1, TYK2, STAT3, p-STAT3, Caspase3, Bax, and Bcl-2.

**Results:**

Th-22 and IL-22 expressions were higher in the CS group than those in the Air group. Both the AG490 and Fezakinumab groups had reduced levels compared with the CS group but remained higher than the Air group. Similarly, IL-22R1 expression was higher in the CS group than in the Air group, while both AG490 and Fezakinumab groups exhibited lower expression than the CS group. H&E staining indicated less severe airway remodeling, alveolar enlargement, and inflammatory cell infiltration in the AG490 and Fezakinumab groups compared with the CS group. Western blot analysis revealed higher levels of JAK1, p-STAT3, and Caspase3 and lower levels of STAT3 and Bcl-2 in the CS group than those in the Air group. Both AG490 and Fezakinumab groups exhibited lower levels of JAK1, p-STAT3, and Caspase3 than the CS group, while Fezakinumab groups exhibited lower levels of STAT3 and higher levels of Bcl-2 than the CS group.

**Conclusions:**

Th-22 cells and IL-22 play crucial roles in COPD pathogenesis. Fezakinumab and AG490 mitigate airway remodeling, alveolar enlargement, and pulmonary tissue inflammatory cell infiltration by modulating JAK/STAT pathways and apoptosis. These results suggest promising targeted therapies for COPD, potentially improving patient outcomes by addressing inflammatory mechanisms.

## 1. Introduction

Chronic obstructive pulmonary disease (COPD) is a prevalent and debilitating respiratory condition characterized by persistent respiratory symptoms and airflow limitation. According to the World Health Organization, COPD ranks as the third leading cause of death worldwide, affecting over 251 million people globally and contributing to more than three million deaths annually [1]. Previous studies have elucidated the pathophysiology of COPD, revealing chronic inflammation and irreversible lung tissue damage as primary factors leading to progressive lung function decline [2]. Recent research has identified several key molecular pathways implicated in COPD pathogenesis, including inflammation, oxidative stress, and apoptosis [3]. Among these, the JAK/STAT pathway has garnered significant attention due to its role in mediating inflammatory responses and cellular apoptosis. Specifically, the JAK1/STAT3 axis has been implicated in regulating proinflammatory cytokines and apoptotic markers critical for COPD development and progression [4]. Current therapeutic strategies, such as bronchodilators and anti-inflammatory agents, offer symptomatic relief but fail to arrest disease progression or reverse lung damage [5], emphasizing the pressing need for novel therapeutic approaches targeting underlying disease mechanisms.

The involvement of Th-22 cells and their associated cytokine, IL-22, in pulmonary diseases has attracted considerable interest. Th-22 cells, a subset of CD4+ T cells producing IL-22, play a crucial role in mucosal immunity and tissue repair [6–8]. Elevated IL-22 levels have been linked to the pathogenesis of various inflammatory diseases, including psoriasis and inflammatory bowel disease, with IL-22 blockade showing therapeutic promise [9, 10]. In COPD, the implication of Th-22 cells and IL-22 suggests a potential pathogenic link, highlighting the therapeutic potential of modulating this axis [11, 12]. IL-22-deficient mice did not develop cigarette smoke–induced airway remodeling and emphysema-like alveolar enlargement [13]. IL-22 levels significantly increase in the peripheral blood of patients with COPD, crucial for disease development and progression [14, 15]. However, the precise mechanisms by which cigarette smoke induces these molecular changes remain unclear.

Fezakinumab (an IL-22 antibody) has demonstrated therapeutic potential in skin diseases, while AG490 can inhibit the activation of STAT3 by IL-22 [16, 17]. Our study aimed to investigate the therapeutic effects of fezakinumab and AG490 in a cigarette smoke–induced COPD mouse model. This research provides insights into the role of the Th-22/IL-22 axis in COPD and evaluates the therapeutic potential of fezakinumab and AG490, offering hope for improved management of this chronic, intractable disease.

## 2. Material and Methods

### 2.1. Equipment and Reagents

AG490 (Alexis Biomol, USA), fezakinumab (Novartis, Switzerland), Mouse IL-22 Alexa Fluor 647-conjugated Antibody (Bio-Techne, USA), flow cytometer (EC201827C1, USA), FITC fluorescent anti-CD4 antibody (Abcam, United Kingdom), mouse interleukin-22 ELISA detection kit (Nanjing Jiancheng, China), image scanning system with dial scanner (Shenzhen Shengqiang, China), and antibodies (Jak1, Tyk2, STAT1, STAT3, p-STAT3, Caspase3, Bax, and Bcl-2) (Abcam, UK).

### 2.2. Animals

C57BL/6 mice (male, 8–10 weeks old, weighing 18–22 g each) were obtained from the Experimental Animal Center of Kunming Medical University. Mice were acclimatized for 1 week prior to experimentation. They were then randomly divided into four groups (6 mice per group): the Air group exposed to normal air, the CS group exposed to cigarette smoke, the AG490 group which received intraperitoneal injections of AG490 (10 mL/kg, 10 μmol/L, once a day) one hour prior to cigarette smoke exposure in the morning for 8 weeks, and the Fezakinumab group which received intraperitoneal injections of fezakinumab (5 mg/kg, 1 μg/mL, once a day) one hour before cigarette smoke exposure in the morning for 8 weeks. Mice were exposed to either ambient air or nose-only inhalation of cigarette smoke (mice were placed in an 80 cm × 60 cm × 30 cm smoke chamber and exposed to Lushan Jingsheng brand cigarettes (tar content 10 mg/cigarette, nicotine content 1.2 mg/cigarette, and carbon monoxide content 12 mg/cigarette) for 3 times a day, with a total of 10 cigarettes smoked each time, with an interval of 2 min between each cigarette, for 5 days a week (Monday–Friday)) for 8 weeks as per established protocols [13, 18].

The mouse breeding environment was maintained at a temperature of 24 ± 2°C and air humidity of 50%–60%. The feed consisted of a conventional feed comprising 11% fat, 29% protein, and 60% carbohydrates.

Following modeling, mice were euthanized via cervical spine dislocation, and the left thoracic cavity was opened. The left main bronchus was ligated upward along the lung lobes; a 22G venous indwelling catheter was inserted, and the trachea was ligated after tracheotomy. A 1-mL syringe connected to the tail of the venous indwelling needle was used to inject sterile PBS. A 5-mL sample of single-sided bronchoalveolar lavage (BAL) was collected, and after PBS injection, the liquid was slowly recovered at low negative pressure and washed thrice, achieving a recovery rate of 90%–92%.

### 2.3. Flow Cytometry

BALF was collected from mice and centrifuged at low speed to remove cell clumps. The total cell count in BALF was suspended in staining buffer and assessed using a hemocytometer. Antibodies specific to surface markers of Th-22 cells were added and incubated at 4°C for 30 min. After washing to remove unbound antibodies, cells were incubated with fixation buffer for 15 min at room temperature, followed by permeabilization buffer and antibodies specific to intracellular markers of Th-22 cells. After a further 30-min incubation at 4°C, cells were washed and analyzed using a flow cytometer with optimized gain settings. At least 10,000 events were collected per sample, and analysis was performed using appropriate software. Th-22 cells were identified based on their surface and intracellular marker expression patterns. The percentage and absolute number of Th-22 cells in BALF were calculated.

### 2.4. ELISA

ELISA plates were prepared as per the manufacturer's instructions, including addition of capture antibody specific to IL-22, detection antibody, substrate solution, and wash buffer. BALF collected from the mouse model was centrifuged to separate cells from supernatant, which was then appropriately diluted. Capture antibody specific to IL-22 was added to the ELISA plate wells and incubated overnight at 4°C. After washing, wells were blocked and incubated with diluted BALF supernatant for 2 h at room temperature. Following further washing, detection antibody specific to IL-22 was applied, incubated, and washed. The detection antibody was used to visualize the antigen–antibody reaction, and absorbance of each well was measured at a wavelength specific to the substrate using a microplate reader. A standard curve generated from reference wells with known IL-22 concentrations was used to determine the IL-22 concentration in the sample.

### 2.5. H&E Staining

Upon excision, the lungs were immersed in a 10% neutral-buffered formalin solution for at least 24 h to preserve tissue morphology. Following fixation, lung tissue underwent dehydration in increasing concentrations of alcohol (70%, 80%, 95%, and 100%) before being impregnated with paraffin wax. The tissue was then embedded in paraffin blocks and cut into thin slices (5–7-μm thick) using a microtome. These sections were mounted on slides and underwent deparaffinization in xylene, followed by rehydration in decreasing concentrations of alcohol (95%, 80%, and 70%) and tap water. Nuclei were stained blue with hematoxylin and cytoplasm pink with eosin, providing contrast for visualizing different cell types and structures in the lung tissue. Stained sections were dehydrated again using absolute alcohol and xylene, covered with a coverslip using a mounting medium, and examined under an image scanning system with dial scanner to observe lung tissue morphology and any pathological changes.

### 2.6. IHC Staining

Lung tissue sections were mounted onto glass slides and fixed with paraformaldehyde. Deparaffinization was performed by immersing slides in xylene and absolute alcohol. Antigen retrieval was achieved by heating slides in citrate buffer (pH 6.0). Slides were then incubated in blocking solution (5% normal serum) before applying the primary antibody specific for IL-22R1 and incubating overnight at 4°C. After washing, a secondary antibody conjugated to a reporter molecule was applied, followed by incubation, washing, and application of a substrate solution for visualizing the antigen–antibody reaction, typically diaminobenzidine (DAB) for color development or a fluorescent substrate for fluorescence microscopy.

### 2.7. Western Blotting

Mouse lung tissue was harvested and homogenized in lysis buffer containing protease inhibitors. The homogenate was incubated on ice for 30 min and then centrifuged at 14,000 rpm for 10 min to remove cell debris. The protein concentration in the supernatant was measured using a protein assay kit or the Bradford assay. Equal amounts of protein were loaded onto a SDS-PAGE gel and electrophoresed at a constant voltage until the dye front reached the bottom of the gel. The separated proteins were transferred from the gel to a nitrocellulose or PVDF membrane using a transfer buffer containing methanol. The membrane was then blocked with 5% nonfat dry milk or BSA in TBST for 1 h at room temperature or overnight at 4°C. After blocking, the membrane was incubated with primary antibodies specific to proteins of interest (Jak1, Tyk2, STAT1, STAT3, p-STAT3, Caspase3, Bax, and Bcl-2) overnight at 4°C. Following primary antibody incubation, the membrane was washed with TBST and then incubated with a secondary antibody conjugated to horseradish peroxidase (HRP) for 1 h at room temperature. After washing, the protein bands were visualized using a chemiluminescent substrate. The membrane was exposed to x-ray film or imaged using a digital imaging system. Band intensity was quantified using image analysis software, and the values were normalized to a loading control (GAPDH) to determine the relative abundance of each protein in the sample.

### 2.8. Statistical Analysis

GraphPad 9.5 software was utilized for data analysis and graph preparation in this study. Data are presented as the mean ± SD. The Student's *t*-test was employed for comparisons between two groups. Additionally, one-way analysis of variance (ANOVA) followed by Tukey's post-test was conducted for multiple comparisons involving three or more groups. Statistical significance was defined as *p* < 0.05.

## 3. Results

### 3.1. Th-22 Cell Expression and IL-22 Levels in BALF

**Figure 1:**
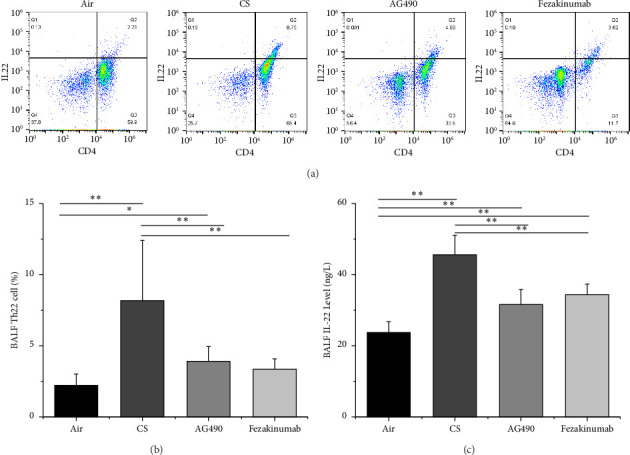
Expression of Th-22 cells and IL-22 levels in BALF. (a-b) Flow cytometry detection of Th-22 cells in the BALF of mice. (c) Measurement of IL-22 expression levels in the BALF of mice by ELISA. ^∗∗^*p* < 0.01 and ^∗^*p* < 0.05. Data are presented as the means ± SD (*n* = 6).

We utilized flow cytometry to detect Th-22 cells in the BALF of mice (Figure 1(a)) and employed ELISA to measure IL-22 levels in the BALF. We observed a significant increase in Th-22 cell content in the BALF of the CS group compared with the Air group (*p* < 0.01), while the Th-22 cell content in the AG490 and Fezakinumab groups was notably lower than that in the CS group (*p* < 0.01) but higher than that in the Air group (Figure 1(b)). IL-22, as an associated cytokine of Th-22 cells, exhibited similar trends in the BALF of mice. IL-22 levels in the BALF of the CS group were significantly higher than those in the Air group (*p* < 0.01). Meanwhile, IL-22 levels in the AG490 and Fezakinumab groups were significantly lower than those in the CS group (*p* < 0.01) but higher than those in the Air group (Figure 1(c)).

### 3.2. Histological Analysis of Lung Tissue

**Figure 2:**
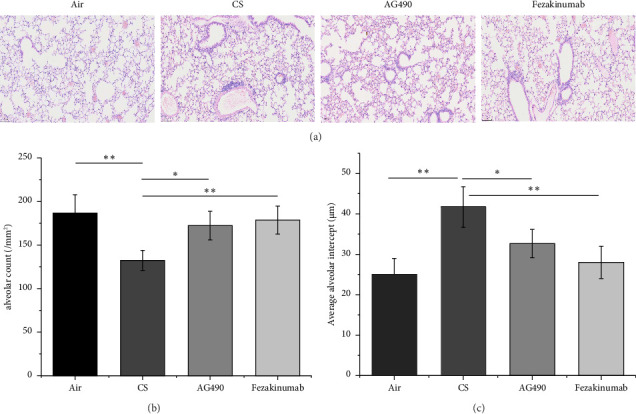
H&E staining for lung tissue. (a) Evaluation of inflammation, airway remodeling, and alveolar size of lung tissues using H&E staining. (b) Verification of average alveolar count. (c) Verification of average alveolar intercept. ^∗∗^*p* < 0.01 and ^∗^*p* < 0.05. Data are presented as the mean ± SD (*n* = 6).

H&E staining was used to observe inflammation, airway remodeling, and average alveolar intercept in mouse lung tissues. The results showed (Figure 2(a)) that in the Air group, the lung tissue structure was intact, with clear staining, uniform average alveolar intercept, no obvious thickening of the pulmonary interstitium, no epithelial cell proliferation or shedding in the small bronchi, and basically no inflammatory cell infiltration. In the CS group, the alveolar structure changed significantly, the number of alveoli decreased, small bronchi epithelium proliferated, and increased accumulation of inflammatory cells was observed around the blood vessels and trachea, some of which were multilayered, with increased thickness of the pulmonary interstitium. In the AG490 group, the pathological changes of lung tissue were still obvious, interstitial thickening was observed, the number of alveoli decreased, and the number of large alveoli decreased compared with the CS group, with obvious inflammatory cell aggregation observed around the bronchi. In the Fezakinumab group, compared with the CS group, the number of alveoli recovered significantly, the average alveolar intercept was relatively uniform, the number of large alveoli decreased, no obvious proliferation of tracheal epithelial cells was observed, a small amount of inflammatory cell infiltration was still observed around the trachea but much less than the CS group, and no obvious thickening of the interstitium was seen. The average alveolar intercept in the CS group was significantly larger than those in the Air group (*p* < 0.01), while the average alveolar intercept in the AG490 group were significantly smaller than those in the CS group (*p* < 0.05), and the average alveolar intercept in the Fezakinumab group were significantly smaller than those in the CS group (*p* < 0.01), but both were larger than those in the Air group (Figure 2(c)). The alveolar count in the CS group was significantly lower than that in the Air group (*p* < 0.01), whereas the alveolar counts in the AG490 group were significantly higher than those in the CS group (*p* < 0.05), and the alveolar counts in the Fezakinumab group were significantly higher than those in the CS group (*p* < 0.01), but both were lower than those in the Air group (Figure 2(b)).

**Figure 3:**
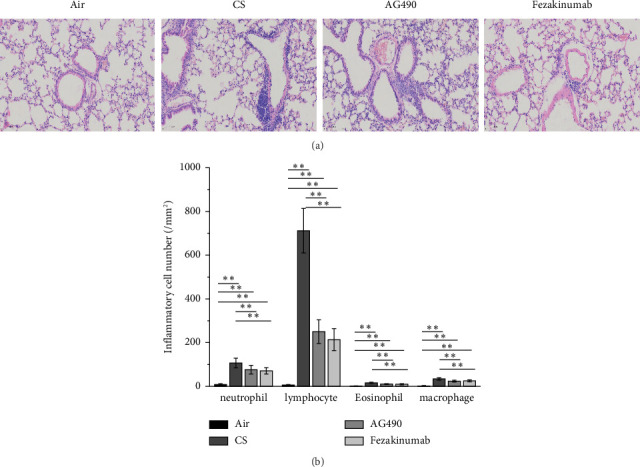
H&E staining for lung tissue. (a) Evaluation of inflammation cell of lung tissues using H&E staining. (b) Verification of neutrophils, lymphocytes, eosinophils, and macrophages. ^∗∗^*p* < 0.01 and ^∗^*p* < 0.05. Data are presented as the mean ± SD (*n* = 6).

We simultaneously compared the infiltration levels of inflammatory cells in the lung tissue of mice (Figure 3(a)). The study indicated that neutrophils, lymphocytes, eosinophils, and macrophages in the lung tissue were significantly higher in the CS, AG490, and Fezakinumab groups compared with the Air group (*p* < 0.01, Figure 3(b)). In contrast, the levels of neutrophils, lymphocytes, eosinophils, and macrophages in the AG490 and Fezakinumab groups were significantly lower than those in the CS group (*p* < 0.01, Figure 3(b)).

### 3.3. Analysis of IL-22R1 Expression in Lung Tissue

**Figure 4:**
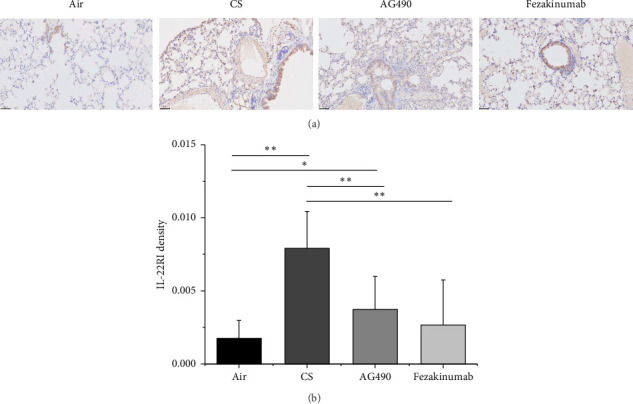
IHC staining for lung tissue. (a) Observation of IL-22R1 expression by IHC. (b) Verification of IL-22R1 expression. ^∗∗^*p* < 0.01 and ^∗^*p* < 0.05. Data are presented as the mean ± SD (*n* = 6).

To explore the role of IL-22 in the COPD mouse model and the protective effects of AG490 and fezakinumab on emphysema, we evaluated the expression of IL-22R1 in mouse lung tissue using immunohistochemistry (Figure 4(a)). In the Air group, IL-22R1 expression was relatively low and mainly distributed in the bronchial epithelium, showing yellowish-brown staining, with less expression in other areas. In the CS group, there was a more obvious IL-22R1 expression visible in the overall field of view, with scattered positive staining observed in the alveolar epithelium and interstitial cells, with both the number and area significantly increased compared with the control group. Compared with the CS group, the AG490 group showed a significant reduction in the number and area of IL-22R1 expression (visualized as brown patches), which was also observed in the tracheal epithelium. The Fezakinumab group, compared with the CS group and AG490 group, showed a reduction in the expression level and area of IL-22R1, with scattered distribution in the alveolar epithelium and brown expression in the tracheal epithelium, showing more significant recovery overall. Quantitative analysis of IL-22R1 revealed that the expression level of IL-22R1 in the lung tissue of the CS group was significantly higher than the Air group (*p* < 0.01); the AG490 group and Fezakinumab group showed significantly lower IL-22R1 expression levels compared with the CS group (*p* < 0.01) but still higher than the Air group (Figure 4(b)).

### 3.4. Protein Expression in Lung Tissue

**Figure 5:**
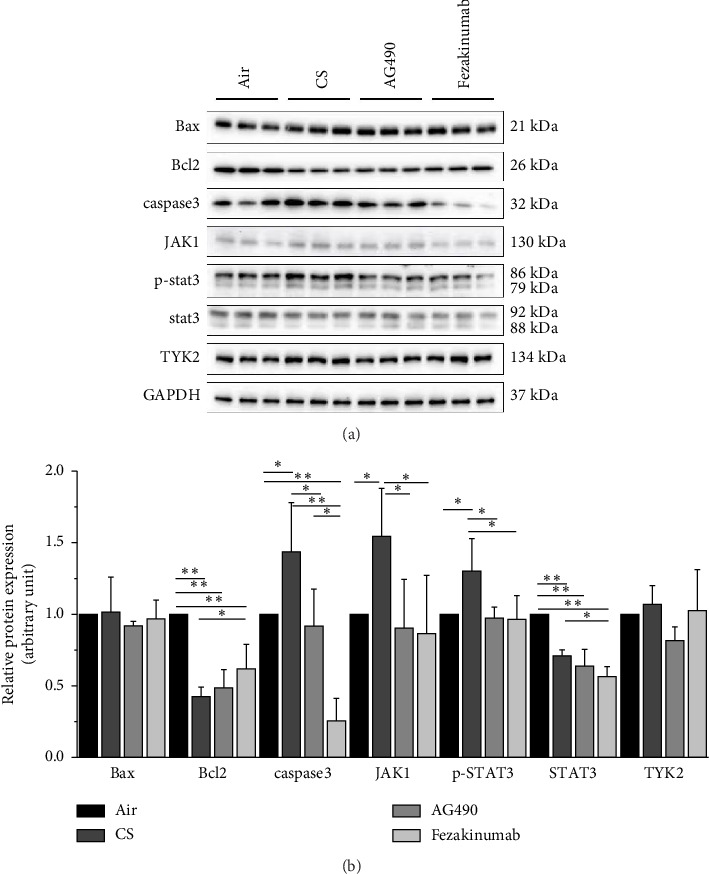
Western blotting for lung tissue. (a-b) Verification of Jak1, TYK2, STAT3, p-STAT3, Caspase3, Bax, and Bcl-2 protein expression by Western blotting. ^∗∗^*p* < 0.01 and ^∗^*p* < 0.05. Data are presented as the mean ± SD (*n* = 3).

To further explore the protective mechanism of AG490 and fezakinumab against cigarette smoke–induced COPD, we used western blotting to detect the expression of related proteins in lung tissue (Figure 5). Compared with the Air group, Bcl-2 and STAT3 expressions in the CS group decreased significantly (*p* < 0.01), while JAK1, Caspase3, and p-STAT3 increased significantly (*p* < 0.05). Compared with the CS group, Caspase3, JAK1, and p-STAT3 in the AG490 group decreased significantly (*p* < 0.05), while Bcl-2 and the STAT3 level showed no significant difference compared with the CS group. Caspase 3, JAK1, STAT3, and p-STAT3 in the Fezakinumab group decreased significantly, while Bcl-2 increased significantly compared with the CS group. There was no significant difference in Bax and TYK2.

## 4. Discussion

COPD represents a significant global health challenge characterized by persistent airflow limitation, primarily caused by long-term exposure to noxious particles or gases, with cigarette smoke being the predominant risk factor. This progressive disease encompasses two main phenotypes: chronic bronchitis and emphysema, both leading to severe respiratory symptoms, reduced quality of life, and increased mortality rates (References [19, 20]). COPD is associated with extensive pulmonary inflammation, structural changes, and alterations in the immune response, which complicate its management and therapeutic strategies (References [21, 22]). Despite the high prevalence and healthcare burden posed by COPD, the underlying mechanisms that contribute to its pathogenesis remain inadequately understood, underscoring the need for targeted research to unravel the complexities of this disease. Recent studies have shown that neutrophil elastase, MMP-12, and mucus production targeting neutrophil infiltration may become new therapeutic targets for COPD (References [23, 24]), while dupilumab and mepolizumab targeting eosinophilic infiltration have shown relatively good efficacy [25, 26].

In this study, we investigate the effects of cigarette smoke on the development of COPD in a mouse model, focusing specifically on the role of TH-22 cells and IL-22 in the pathogenesis of the disease. Previous research has identified various immune cells and cytokines involved in COPD; however, the specific contributions of TH-22 cells and IL-22 remain underexplored. Our findings reveal that cigarette smoke exposure significantly elevates TH-22 cell levels and IL-22 expression, leading to inflammatory responses and lung tissue damage. Additionally, we assess the therapeutic potential of modulating IL-22 signaling pathways through agents such as AG490 and fezakinumab, which may offer novel strategies for managing COPD [21, 22, 27].

The findings of this study provide significant insights into the molecular mechanisms underlying the role of TH-22 cells and IL-22 in the pathogenesis of COPD exacerbated by cigarette smoke exposure. The elevated levels of TH-22 cells and IL-22 in BALF from the CS group suggest a robust immune response, which is consistent with previous research indicating that cytokines play critical roles in inflammatory responses associated with COPD [20]. The observed downregulation of TH-22 cell levels and IL-22 expression in the AG490 and Fezakinumab treatment groups further reinforces the therapeutic potential of targeting IL-22 signaling pathways. By inhibiting IL-22, these agents may mitigate the inflammatory responses that contribute to lung tissue damage and COPD progression, highlighting the importance of TH-22 cells and IL-22 as novel therapeutic targets in managing COPD exacerbations [28, 29].

The histopathological changes observed in lung tissue, including diminished alveolar counts and increased inflammatory cell infiltration in the CS group, substantiate the detrimental effects of cigarette smoke on lung architecture. This is in line with existing literature that correlates structural alterations in lung tissue with functional impairment in COPD patients (References [21, 28]). The partial recovery noted in the AG490 and Fezakinumab groups indicates that therapeutic interventions can reverse some of the tissue damage caused by cigarette smoke, suggesting the potential for these treatments to improve lung function over time. Moreover, the relationship between histopathological changes and clinical outcomes emphasizes the need for effective biomarkers to assess disease severity and treatment response in COPD patients [30].

In terms of the molecular mechanisms, the significant increase in IL-22R1 expression in the CS group points to the potential for IL-22 to drive inflammation and tissue remodeling in COPD (Reference [31]). The reduction of IL-22R1 levels in the treatment groups further underscores the role of IL-22 signaling in mediating these pathological processes. The observed changes in apoptosis-related proteins, including the upregulation of Bcl-2 and downregulation of Caspase3 in treated groups, suggest a protective mechanism against inflammation-induced cell death, thereby preserving lung function [32]. Overall, these findings elucidate a complex interplay between TH-22 cells, IL-22, and various signaling pathways, reinforcing the need for further exploration of these mechanisms to develop effective therapeutic strategies for COPD management.

The primary limitations of this study arise from the lack of pulmonary function assessments due to funding and equipment constraints, which may hinder the comprehensive evaluation of therapeutic efficacy in a clinical context. Additionally, the small sample size, with six mice per experimental group, raises concerns about the statistical power and generalizability of the findings. The Western blotting results reported as “*n* = 3” were constrained by budget limitations, although this number is generally considered sufficient for preliminary statistical validation. Another limitation of this study is its reliance on a murine model to simulate cigarette smoke–induced COPD. While this model offers valuable insights into pathophysiological mechanisms and potential therapeutic targets, it may not fully replicate the complexity of COPD in humans. Furthermore, the focus on specific molecular markers, such as IL-22, IL-22R1, the JAK/STAT pathway and apoptosis-related proteins, although informative, may overlook other critical pathways and cellular interactions involved in COPD progression.

Our findings demonstrated that both AG490 and fezakinumab significantly mitigated the pathological changes associated with cigarette smoke–induced COPD in mice. These interventions effectively reduced the levels of Th-22 cells, IL-22, and IL-22R1 expression and modulated key components of the JAK/STAT signaling pathway. Notably, fezakinumab showed a more pronounced effect in restoring lung architecture and reducing inflammation than AG490. These results suggest that targeting the IL-22/IL-22R1 axis and JAK/STAT pathway holds promise for the development of novel therapeutic strategies for COPD. However, further research is needed to validate these findings in humans and explore the long-term efficacy and safety of these potential treatments.

## Data Availability

The data of this study are available on request from the author Yuwei Wang (Email: 18281701380@163.com).
